# Persistent Chaos of Measles Epidemics in the Prevaccination United States Caused by a Small Change in Seasonal Transmission Patterns

**DOI:** 10.1371/journal.pcbi.1004655

**Published:** 2016-02-04

**Authors:** Benjamin D. Dalziel, Ottar N. Bjørnstad, Willem G. van Panhuis, Donald S. Burke, C. Jessica E. Metcalf, Bryan T. Grenfell

**Affiliations:** 1 Department of Ecology and Evolutionary Biology and Woodrow Wilson School of Public and International Affairs, Princeton University, Princeton, New Jersey, United States of America; 2 Department of Biology, Pennsylvania State University, University Park, Pennsylvania, United States of America; 3 Department of Epidemiology, University of Pittsburgh, Pittsburgh, Pennsylvania, United States of America; 4 Graduate School of Public Health, University of Pittsburgh, Pittsburgh, Pennsylvania, United States of America; 5 Fogarty International Center, National Institutes of Health, Bethesda, Maryland, United States of America; Imperial College London, UNITED KINGDOM

## Abstract

Epidemics of infectious diseases often occur in predictable limit cycles. Theory suggests these cycles can be disrupted by high amplitude seasonal fluctuations in transmission rates, resulting in deterministic chaos. However, persistent deterministic chaos has never been observed, in part because sufficiently large oscillations in transmission rates are uncommon. Where they do occur, the resulting deep epidemic troughs break the chain of transmission, leading to epidemic extinction, even in large cities. Here we demonstrate a new path to locally persistent chaotic epidemics via subtle shifts in seasonal patterns of transmission, rather than through high-amplitude fluctuations in transmission rates. We base our analysis on a comparison of measles incidence in 80 major cities in the prevaccination era United States and United Kingdom. Unlike the regular limit cycles seen in the UK, measles cycles in US cities consistently exhibit spontaneous shifts in epidemic periodicity resulting in chaotic patterns. We show that these patterns were driven by small systematic differences between countries in the duration of the summer period of low transmission. This example demonstrates empirically that small perturbations in disease transmission patterns can fundamentally alter the regularity and spatiotemporal coherence of epidemics.

## Author Summary

Measles epidemics continue to pose a significant public health risk wherever vaccination coverage is low. In such populations transmission rates tend to fluctuate seasonally, mirroring patterns of human aggregation, due to the timing of school terms, and/or the migration of workers and their families. Here we show empirically that slight changes in the seasonal pattern of measles transmission can lead to massive shifts in the complexity of measles dynamics, in some cases driving epidemic patterns that resemble deterministic chaos. Our analysis is based on a comparison of 20-year biweekly measles incidence time series in 80 major cities in the prevaccination era United States and United Kingdom. The results are important in two ways: first, in contrast to previous theory, we show that subtle shifts in seasonal patterns of transmission can cause deterministic chaos in the epidemic dynamics of acute immunizing infections; second, we demonstrate that this new route to deterministic chaos is significantly more robust to stochastic extinction compared with previous chaotic models, suggesting chaotic dynamics may be more common in natural populations than previously thought.

## Introduction

Acute immunizing infections remain a leading cause of death worldwide, and have accounted for a significant portion of all morbidity and mortality throughout human history, especially among children and in countries without adequate vaccination coverage [1–4]. Understanding the processes that determine epidemic patterns in these diseases can aid in forecasting and improve the efficacy of public health interventions. Studying the epidemiological dynamics of these diseases also provides a unique window on population-level predictability and its limitations, in an important applied context.

Epidemics of acute immunizing infections often occur in predictable cycles[5–10]. The underlying drivers of measles epidemics are particularly well understood, consisting of the basic demographic clockwork of repeated depletion of the susceptible population by infection or vaccination, followed by susceptible recruitment through birth. Cycles of human aggregation from school holidays or the migration of workers and their families cause seasonal fluctuations in transmission to sustain recurrent epidemics [2,11]. This overall clockwork is modulated by secular variation in susceptible recruitment caused by changes in birth rate and vaccination uptake [12], and by demographic stochasticity and local extinction in small populations, which predisposes smaller towns and cities to be entrained to the dynamics of larger metropolitan centers [13,14]. Simple mathematical models that incorporate these drivers have in many cases successfully predicted incidence patterns, making measles a canonical system in the study of non-linear population dynamics and prime target for elimination [5,6,11,12,14].

The most intensively studied incidence patterns for measles are from Europe—notably the UK—during the prevaccination era and are characterized by stable limit cycles (regularly occurring seasonal epidemics) with annual or biennial periods [5,11,15,16]. In the biennial cycles, susceptible depletion in the major epidemic years is replenished by births throughout the following year, during which minor epidemics may occur[17]. Increasing birth rate in this context causes the susceptible population to replenish more rapidly, leading to a collapse from biennial to regular annual cycles, as observed during the post-World War II “baby boom” [5,12,18].

In contrast, recent analyses of measles in western Africa—notably Niger—have revealed complex dynamics, featuring episodic epidemics with highly variable amplitudes. These are primarily caused by sharp seasonal increases in population density driven by collective migration [2] resulting in deep epidemic troughs, through nonlinear resonant feedbacks [6,19]. Owing to the intense seasonality, the equations describing measles dynamics in Niger produce deterministically chaotic trajectories, as conjectured by previous theory [6,18]. However, the deep post-epidemic troughs invariably break the chains of transmission, precluding local persistence.

Previous case studies, therefore, suggest an impossible tension with respect to chaos in real world epidemics. Despite its mathematical plausibility [20–23], the large amplitude seasonal fluctuations in transmission rates that have been presumed a prerequisite for chaos [12,17,19], in practice result in so deep epidemic troughs that frequent stochastic extinctions are inevitable[19]. Thus exotic nonlinear dynamics and local persistence have been thought to be in opposition in nature [24]. We refute that hypothesis here by showing evidence of widespread persistent chaos in the epidemic dynamics of prevaccination measles in the United States, which emerged via a new route to chaos that is less prone to stochastic extinction.

## Methods

We take a comparative approach, analyzing 20-year biweekly time series data on measles incidence in 80 major cities, 40 in the US and 40 in England and Wales (UK). To compare these contexts, we fit a Time-series Susceptible Infected Removed (TSIR) model to the measles incidence data for each of the 80 cities [5,6,11]. The TSIR model describes macroscale properties of the stochastic branching process of measles spread, focusing on the expectation for number of secondary cases arising from the current population of infected individuals (hereafter the “deterministic skeleton”) and the probability distribution describing variation around that expectation due to the stochastic nature of infectious disease spread.

Representing the number of infected individuals in generation *t* by *I*_*t*_, the concurrent number of susceptible individuals by *S*_*t*,_ and the population size by *N*_*t*_, the TSIR model is given by

$$E\left[I_{t+1}\right]=\beta_{t}I_{t}^{\alpha }S_{t}N_{t}^{-1}$$ (1)

$$I_{t+1}∼Neg.Bin.\left(E\left[I_{t+1}\right],I_{t}\right)$$ (2)

where *E[*.*]* is the expected value and *β*_*t*_ represents seasonally fluctuating transmission rate in each city. The mixing parameter α, usually set at slightly less than unity, accounts for latent inhomogeneity in contact patterns between susceptible and infected individuals [23], as well as compensating for instabilities arising from discretizing the underlying continuous-time process [25]. Following previous work [11,16], we use α = 0.975 for all cities, which leads to good performance under forward simulation of the model.

The TSIR model for measles operates at the characteristic two-week serial interval of infection. Eq 2 represents the birth-and-death stochasticity inherent in transmission dynamics resulting in a negative binomial distribution of new cases with mean *E[I*_*t+1*_*]* and dispersion parameter *I*_*t*_, so that the variance in *I*_*t+1*_ is given by *E[I*_*t+1*_*]* + *E[I*_*t+1*_*]*^*2*^*/I*_*t*_. To study the deterministic skeleton of the dynamics, we model *I*_*t+1*_ *= E[I*_*t+1*_*]* in place of Eq (2).

Susceptible dynamics are modeled as

$$S_{t+1}=S_{t}+B_{t}-I_{t+1}$$ (3)

where *B*_*t*_ is the observed time-varying birth rate in a given city (see below). Secular variation in susceptible recruitment is a well-known driver of variation in measles periodicity [12] that we account for by using data on birth rates for each city when fitting and doing forward simulations. The full procedure for fitting the TSIR model to data follows well established techniques [5,11] that also included here as Supporting Information (S1 Text).

We assembled biweekly time series of measles incidence in US cities using the Project Tycho database [26] and took biweekly measles incidence and demographic data for cities in the UK from previous work [11,27]. For US cities we took estimates of population size for each city over the period of the study from census data [28] and estimated effective birth rates by differencing biweekly time series of the number of children under one year old [29], adjusting for the rate at which children age out of this class. For total and infant population sizes in the US, biweekly time series were obtained by evaluating at each biweek a spline function fitted to the decennial data (see S1 Text). Variations in the approach to reconstructing US recruitment rate, including varying background infant mortality, and changing the degrees of freedom in spline fitting, did not affect the results.

We used data for the 40 US cities in the Project Tycho database with the most records of measles incidence, which included most major US cities. While the Project Tycho database has measles incidence data from 1903 to 1953, data coverage was uniformly high for these 40 cities between 1920 and 1940, so we used that period in the analysis. For the England and Wales measles data we used the city of London plus the largest 39 cities that were more than 50km from London to prevent a “borough effect” where UK cities in the greater London area are entrained to its dynamics.

Due to limitations on data availability, the US measles data we used extends from 1920–1940, whereas the England and Wales measles data extends from 1944 to 1964. Our analysis accounts for demographic differences associated with the changing time window between the US and the England and Wales data, including differences in birth rates over time among cities and countries. Consequently, the temporal mismatch between the US and UK data does not drive the observed epidemic patterns—evidence from other sources clearly shows that measles epidemics in London, UK and other major UK cities remained predominantly biennial and non-chaotic in the period covered by the US data (1920–1940; see S1 Text)[15,30].

## Results

**Fig 1:**
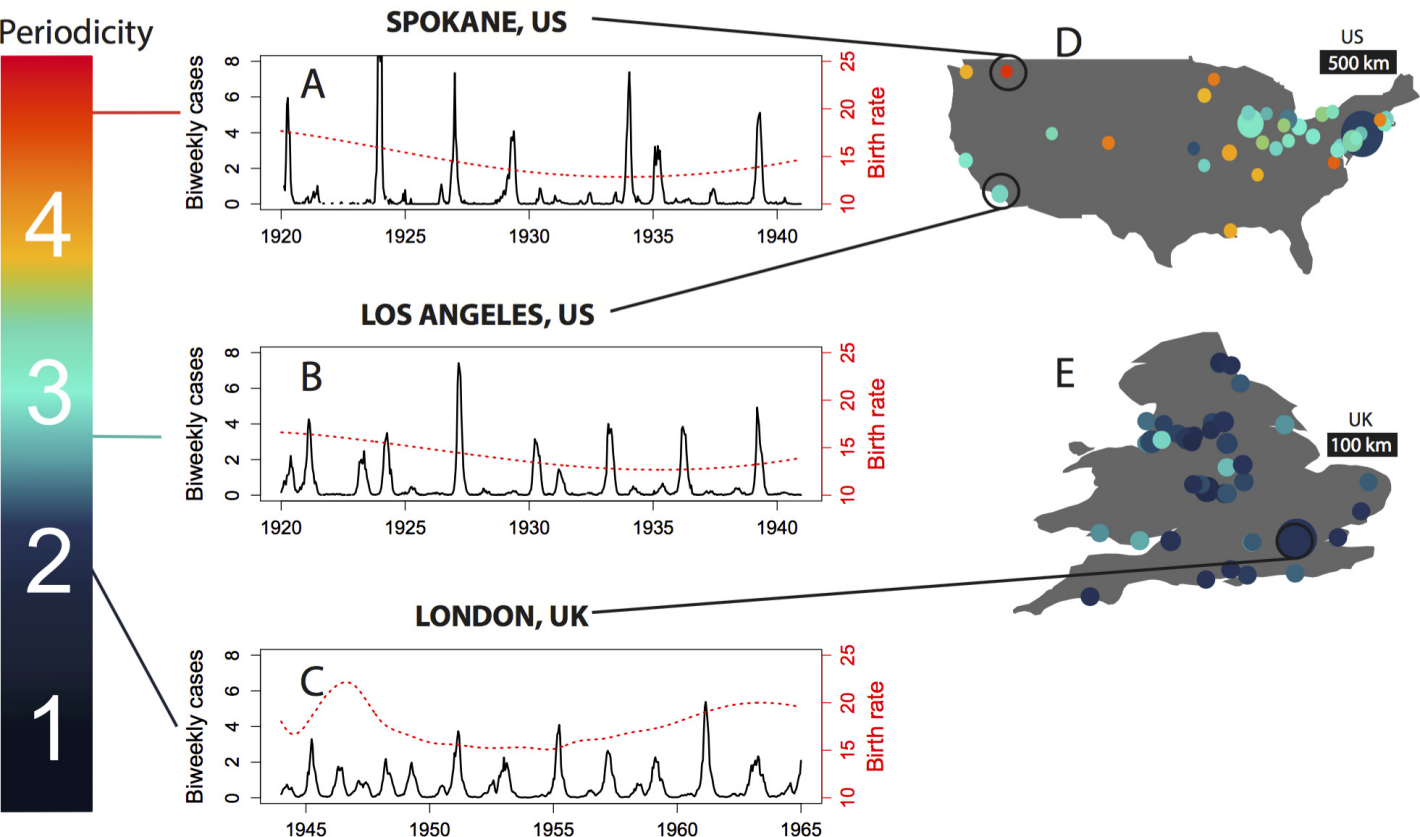
Differences among cities in the amplitude and frequency of historical measles cycles—epidemics in US cities were larger and more episodic than in England and Wales, where outbreaks were predominantly biennial. (A-C) Biweekly incidence of measles per thousand population, corrected for underreporting, for three representative cities in the US (1920–1940) or UK (1944–1964). Red dashed lines show births per thousand population per year. The mean periodicity of each time series is indicated on the left colorbar. (D-E) Location and periodicity of each city in the data, with the area of each circle proportional to mean population.

Measles dynamics varied systematically among cities and countries in the prevaccination era, with US cities exhibiting more diverse and episodic epidemics than cities in the UK. Whereas measles dynamics in the UK were predominantly locked on a biennial cycle, as previously reported [5,6,11,31], a majority of US cities showed lower frequency, higher amplitude oscillations (Fig 1). Consequently, the mean periodicity of a city’s measles incidence (see S1 Text) varied more widely among US cities, and was higher on average, compared to cities in the UK.

**Fig 2:**
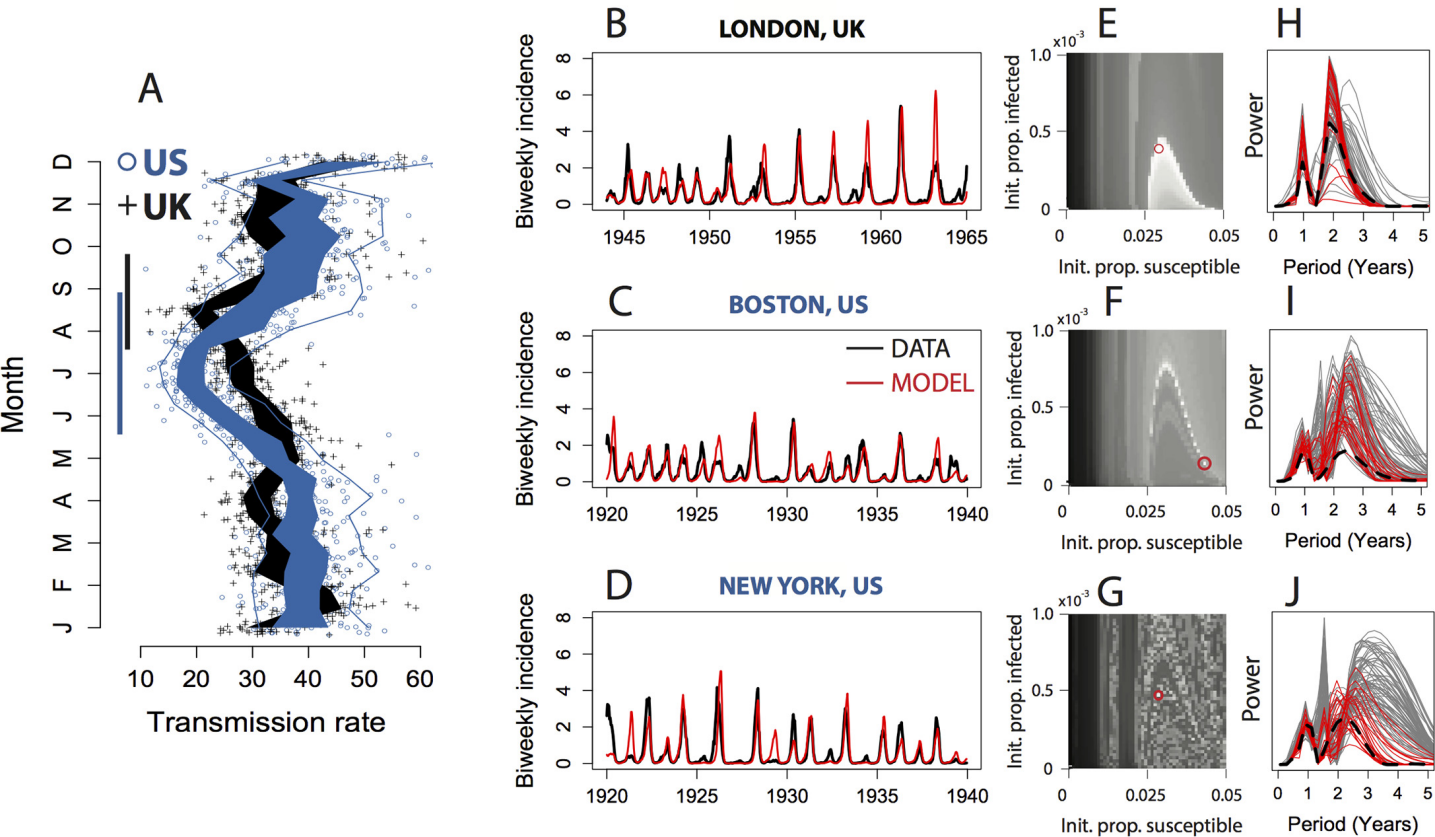
(A) Biweekly transmission rate in 80 cities: 40 in the US (circles) and 40 UK (crosses). Polygons enclose the interquartile range for the transmission rates of cities in each country (US, blue; UK, black), illustrating that systematic differences between countries exceed inter-city differences within a country. Enclosing lines show the 5^th^ and 95^th^ analogous quantiles. Bars on the vertical axis highlight differences among countries in the duration and timing of the summer period of reduced transmission. (B-D) Successful forward simulation of the deterministic skeleton of the TSIR model parameterized with city-level transmission data (red lines) compared to incidence data (black lines) for London, Boston and New York. Simulations were started with the initial conditions indicated by red circles in adjacent panes and run forward for 20 years without further information added from the incidence data. (E-G) performance of the forward simulations starting from different initial conditions. Lighter shades indicate a better fit (measured as sum of squared distance between the model and the data during the peak month of May). (H-J) Periodograms of 100 stochastic simulations of the TSIR model for each of the same three cities, contrasting the periodogram of the data (dashed line) with the best 20 simulations shown in red and the remaining 80 shown in grey.

We found a comparable systematic variation in the shapes of the underlying seasonal transmission patterns in each country, particularly a systematically lengthening of the summer period of low transmission in US relative to the UK (Fig 2A). Given the biology and demography of measles transmission, it is likely that this lengthening is associated with historical differences in timing and duration of school summer holidays between the two countries [11,32]. Corroborating historical data on the timing of school holidays in US cities is not currently available. Whatever their origins, we show that these systematic differences in transmission rates caused measles dynamics in the US to diverge from the stable annual or biennial limit cycles previously characterized in measles epidemics for the UK. US measles cycles exhibit higher and more variable mean periodicity (Fig 2B–2E) and are more sensitive to initial conditions (Fig 2E–2G), which are hallmarks of complex population dynamics[33]. This conclusion is supported by several lines of evidence, as follows.

First, twenty-year forward simulations of the deterministic skeleton of the TSIR model parameterized with the fitted seasonal transmission function for each city yielded close fits to the times series of measles incidence (Fig 2B–2D and Fig C in S1 Text), confirming that observed differences among cities and countries in epidemic complexity can be explained by systematic variation in seasonal transmission patterns. Occasional discrepancies between the data and the forward simulations (such as for London in 1964, where the epidemic was smaller than predicted) are due in part to the accumulation over many biweekly timesteps of measurement errors in the data on birth rates and case counts, which were imperfectly reported. In addition, latent processes not included in the TSIR model, such as variation in age structure, may cause discrepancies between the forward simulations and the data. However, previous work [12] has shown that the long-term impact of such latent structure may be encoded in the shape of the seasonal transmission function, which could explain how simple models can successfully capture the key features of epidemics in complex populations, as is the case with the measles periodicity described here.

As a second line of evidence for transmission-driven differential complexity in US measles epidemics, the deterministic TSIR simulations of measles in US cities were much more sensitive to initial conditions relative to UK cities. That is, slight changes to the initial proportion of the population that was susceptible or infected in US cities produced large differences in epidemic periodicity, but this was not the case for UK cities (Fig 2E–2G). The fine-scale dependence on initial conditions in US cities precludes long-range historical forecasts of measles epidemics in the prevaccination US because the outcome of such simulations depends on precise estimates of the initial proportion of the population that is susceptible and infected, which cannot be estimated without significant statistical uncertainty (Fig 2F and 2G). This is in contrast to the UK, where accurate forecasts of the prevaccination era incidence time series can be achieved from a wide range of starting conditions, making forecasts in the UK resilient to statistical uncertainty in the initial susceptibility of the population (Fig 2E).

Third, the stochastic model (Eq 2), which continually pushes epidemic trajectories away from the deterministic skeleton, provides further evidence of how cities with subtly different seasonal transmission patterns respond to perturbations in the number susceptible and infected. The stochastic simulations also show good correspondences between the model and the data, as measured by assessing whether the distribution of periodograms generated under repeated forward simulation of the stochastic model qualitatively matched the periodogram of the data. The distributions of periodograms for UK cities (Fig 2H) were less dispersed than in the US (Fig 2I and 2J). For US cities, stochastic simulations revealed multiple distinct periodic patterns. These distinct patterns coexist in the same parameter space, emerging as a result of stochastic variation in the simulation process alone (Fig 2I and 2J). In this case the data match a subset of the possible periodograms, while other periodograms suggested by the model for a given parameterization were not observed (Fig 2J). In the parlance of dynamical systems theory this suggests the presence of coexisting attractors[12] or important unstable manifolds [34,35]. In practical terms, the stochastic simulations imply that if time could be repeatedly wound back and played again from a similar starting point, biennial measles dynamics in UK cities would still be biennial, whereas US cities would display a diversity of possible trajectories. Where it happens that measles cycles in a US city are predominantly biennial (e.g. New York), the regular periodicity belies a sensitivity to initial conditions.

**Fig 3:**
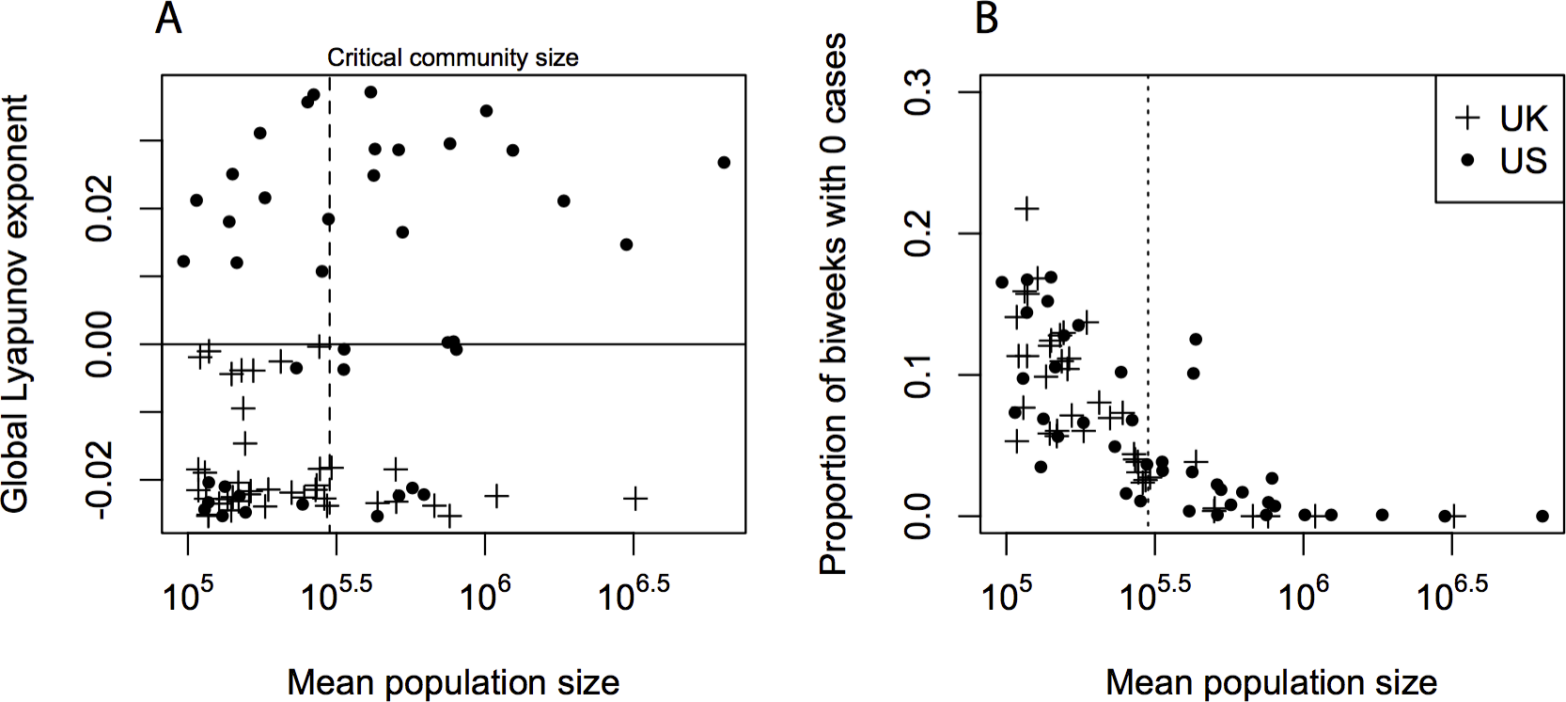
(A) Global Lyapunov exponents estimate the rate of divergence of local dynamics following small perturbations, indicating that the majority of US cities above the critical community size experienced sensitive dependence on initial conditions—the defining feature of chaotic population dynamics. (B) Rates of stochastic local extinction are, however, similar for cities in the UK and US, with extinction unlikely above the common critical community size of approximately 300,000.

As further evidence for chaotic measles dynamics in the US we calculated dominant Lyapunov Exponents (LE; see S1 Text) for each city in the data. LEs measure the rate at which similar epidemic trajectories converge (LE < 0) or diverge (LE >0) [6,36], quantifying the sensitivity of a dynamical system to small changes in state. While all LEs for cities in England and Wales were negative, the majority of US cities had positive LEs (Fig 3A). This corroborates the results of the stochastic simulations, providing another line of evidence for sensitive dependence on initial conditions across US cities, due to a slight change in the shape of the seasonal transmission function relative to cities in the UK.

Surprisingly, however, the resulting complex dynamics in the US were as stable as those in the UK in terms of the risk of local extinction, with local extinction rare in all cities above around 300,000 inhabitants in both countries (Fig 3B). The highly irregular measles dynamics of the US are, thus, as robust to stochastic extinction as the clock-like regularity of the epidemics in the UK. This is surprising because previous models of chaotic epidemic dynamics for seasonally immunizing infections predicted that increased complexity is accompanied by increased risk of stochastic extinction, apparently precluding persistent deterministic chaos in real-world scenarios [12,19,24].

**Fig 4:**
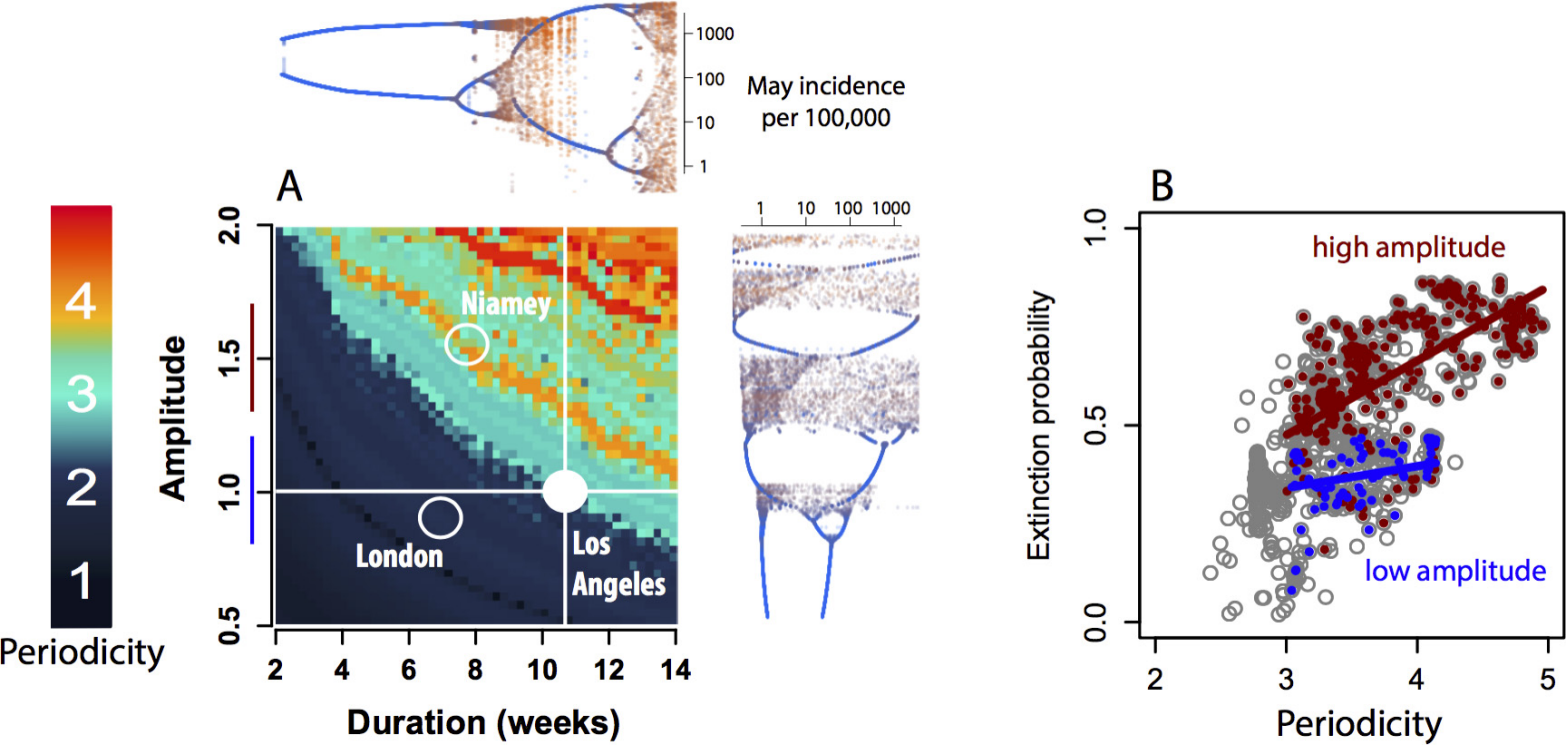
(A) Bifurcation plot for the deterministic skeleton of the seasonally forced TSIR model parameterized for Los Angeles, US, as a function of the duration of the period of low transmission (horizontal axis) and the relative amplitude of seasonal fluctuations (vertical axis) (see S1 Text). Colors indicate the mean periodicity of the resulting dynamics, showing two distinct routes to deterministic chaos. Marginal plots show the predicted number of cases in Los Angeles each May, as seasonal amplitude and duration of the seasonal low-transmission period are varied one at a time. Hollow circles indicate the approximate locations of London, UK, and Niamey, Niger on the bifurcation plot. Simulations were run for 100 years following 100 years of burnin to remove transient dynamics. (B) Proportion of biweeks with <1 case for each of the simulations shown in the main bifurcation diagram (grey circles), for simulations with seasonal transmission amplitudes near 1.0 and with positive Lyupanov exponents (blue circles, and blue line on the vertical axis of the adjacent subfigure), and for simulations with seasonal transmission amplitudes near 1.5 and with positive Lyupanov exponents (red circles, and red line on the vertical axis of the adjacent figure).

Analysis of the clockwork underlying these epidemic dynamics reveals two distinct routes to deterministic chaos for seasonally modulated immunizing infections (Fig 4). To demonstrate these routes we began with the TSIR model for Los Angeles, US, which has a mean periodicity of ~3 years and a positive LE, and systematically varied the amplitude of the seasonal transmission function, and/or the duration of the period of low transmission (see S1 Text), while holding susceptible recruitment constant. On one hand, increasing the amplitude of seasonal oscillations in transmission leads to chaotic dynamics through the previously well-characterized route [6], corresponding to the extinction-prone measles dynamics observed in Niger, where deep epidemic troughs frequently break local chains of transmission [19]. On the other, increasing the duration of the seasonal period of low transmission, while holding seasonal amplitude constant, also leads to chaos. In contrast to the chaotic measles epidemics previously described in Niger, the new route to chaos revealed in the prevaccination US is associated with local persistent chains of transmission (Fig 3B). Therefore, although these distinct routes to chaos yield equivalent levels of deterministic complexity, they are associated with contrasting properties of local persistence: only the new low-amplitude route to chaos exemplified by measles in US cities can sustain true chaotic fluctuations for a significant period of time (Fig 4B).

The existence of distinct routes to chaos with contrasting probabilities of local extinction explains both the complexity and persistence of measles epidemics in the prevaccination United States, as well as the systematic differences between measles epidemics in the prevaccination US, the prevaccination UK and in present-day sub-Saharan Africa. In particular, we found transitions to complex epidemic dynamics do not necessitate high-amplitude fluctuations in transmission rates nor broad secular changes in susceptible recruitment, as previously thought [12]. The US analysis shows that subtle shifts in seasonal transmission patterns can also lead to chaos. But the origins of dynamic complexity—whether through the canonical routes or the newly described low-amplitude route operating in the US—have important implications for the local persistence of the resulting epidemics.

Finally, we note that systematic differences in dynamics at the city level may have propagated to affect countrywide patterns in the spatiotemporal coherence of disease incidence patterns. While the annual or biennial predictable measles cycles in UK cities represented synchronous phase locked oscillations across the entire island[14,37], the locally persistent chaotic dynamics in the US appeared to break the phase-lock in US measles epidemics, both over the same spatial scale as the UK, and overall (Fig B in S1 Text). However, further, more detailed, spatial analyses are necessary to tackle systematic variation in the strength of these correlations at inter-city and regional scales. This may be an interesting avenue for future work.

## Discussion

The realization in the 1970s that simple models of population growth can have complex dynamics [20], spurred several decades of effort in ecology and epidemiology to explain highly variable time series using a few general equations [22,38], with hopes of emulating the success of Newtonian physics [24]. Although controlled laboratory experiments supported the hypothesis that complex dynamics in living populations can emerge from simple rules [39] applications to real-world scenarios were often stymied by the role of stochasticity—chance events play a significant role in the growth trajectories of many live populations, but such variation is minimized in deterministic models and controlled experiments.

A particular challenge was the fact that canonical chaotic models of seasonally forced epidemics carried a high risk of stochastic extinction. Specifically, the route to chaos described by these models involves broad-scale changes in susceptible recruitment, such as changes in birth rate or vaccination coverage, or significant structural changes in the seasonal pattern of transmission, such as changes in the amplitude of seasonal fluctuations in transmission rate [12,19]. But these structural changes result in deep epidemic troughs, where the chain of transmission is maintained by only a few individuals. This greatly increases the likelihood that an epidemic will fade out, due to random variation in the timing of infection and removal events [12,19,40]. This tradeoff, where achieving a realistic level complexity requires an unrealistic rate of stochastic fadeouts, apparently precluded persistent deterministic chaos as an explanation for capricious incidence time series. In contrast, we have shown that small shifts in the seasonal pattern of disease transmission can offer a new, more stable, route to persistent deterministic chaos (Fig 4).

The relative importance of noise and determinism in population dynamics varies with context: for instance, stochasticity appears to generate proportionally more of the amplitude in rubella cycles (as well as driving patterns in local extinction), because the deterministic skeleton of these dynamics falls on an attractor that is globally less stable [41]. Similarly, the intermittent 3–4 year periodic pertussis dynamics is thought to emerge from stochastic resonance around a deterministic skeleton with a dominant annual period [42]. For US measles dynamics in the prevaccination era, the effects of stochasticity and determinism are inextricably intertwined through highly nonlinear sensitive dependence on initial conditions.

Although not conclusive, analysis of cross correlation in measles incidence across cities suggests that US cities may have had less synchronized epidemics at regional and country-wide scales (Fig B in S1 Text). The level of synchrony among connected populations has been shown to influence patterns of disease persistence *per se*, but predictions for the impact of chaos on metapopulation dynamics have been somewhat equivocal, as epidemic chaos has its own complex relationship with persistence. Specifically, spatial decorrelation, such as that seen among US cities, can improve disease persistence in a metapopulation context, as subpopulations that experience local extinctions may be more likely to be rescued by connected subpopulations that have not experienced a fadeout—the metapopulation “rescue effect” [43–45]. However, spatial decorrelation specifically linked to chaos was previously thought to be an unlikely source of pathogen persistence, because deep seasonal troughs in transmission rates, thought to be a prerequisite to chaos, tend to synchronize the timing of fadeouts across the metapopulation, diminishing the rescue effect [40]. The new route to locally persistent but decorrelated dynamics may change this perspective.

Our analysis demonstrates the impacts of chaos on the metapopulation dynamics of cities, showing that the network consequences of complex epidemic patterns depend on the origins of the complexity. On one hand, if complex cycles emerge via high amplitude fluctuations in transmission rates, then local populations will be more likely to experience synchronous fadeouts, and metapopulation rescue effects will not considerably improve local persistence[19,40]. On the other hand, if complex epidemic cycles emerge from slight changes in the duration of the seasonal period of low transmission—as shown here for measles in the prevaccination era US—local populations will experience relatively higher rates of disease persistence, in addition to a plausibility of significant metapopulation rescue effects. The enhanced persistence is consistent with the data presented here, where the observed probability of measles fadeouts across US cities (Fig 3B) was still lower than that predicted in single-city simulations under the more stable route to chaos operating in the US, suggesting the presence of a rescue effect[44] (Fig 4B).

In conclusion, the emergence of persistent chaotic epidemics in the prevaccination US from small shifts in seasonal transmission patterns reveals a novel and potentially widespread route to chaos in population dynamics[24,46,47]. Moreover, these results show empirically that the viability of chaotic populations depends subtly on the route to chaos. In practice, this means that small perturbations in transmission rates, such as those caused by shifts in host behavior or the imposition of epidemic control measures, can lead to a rapid erosion of the capacity to forecast epidemic patterns, which can in turn reduce the efficacy of control strategies such as reactive vaccination[2,19,48,49]. Generally, population dynamics are deterministically more sensitive to perturbations than previously thought.

## Supporting Information

S1 Text Supplementary text, including additional information on data sources and methods, and supporting figures. (DOCX)

S1 Table Biweekly transmission rates in 80 cities in the US and UK. (CSV)
