# Supplementary material for: Persistent Chaos of Measles Epidemics in the Prevaccination United States Caused by a Small Change in Seasonal Transmission Patterns
Source: PLoS Comput Biol. 2016 Feb 4;12(2):e1004655. doi: 10.1371/journal.pcbi.1004655 (PMC4741526; doi:10.1371/journal.pcbi.1004655)
Supplement: S1 Text — (DOCX) [file pcbi.1004655.s001.docx]

**Supporting Information**

*Data*

We assembled biweekly time series of measles incidence in US cities using the Project Tycho database [26] and took biweekly measles incidence and demographic data for cities from England and Wales from previous work [11,27]. For US cities we took estimates of population size for each city over the period of the study from census data [28] and estimated effective birth rates by differencing biweekly time series of the number of children under one year old [29], adjusting for the rate at which children age out of this class. For total and infant population sizes in the US, biweekly time series were obtained by evaluating at each biweek a spline function fitted to the decennial data. Variations in the approach to reconstructing US recruitment rate, including varying background infant mortality, and changing the degrees of freedom in spline fitting, did not affect the results.

We used data for the 40 US cities in the Project Tycho database with the most records of measles incidence, which included most major US cities. While the Project Tycho database has measles incidence data from 1903 to 1953, data coverage was uniformly high for these 40 cities between 1920 and 1940, so we used that period in the analysis. For the England and Wales measles data we used the city of London plus the largest 39 cities that were more than 50km from London (Table S1), to prevent a “borough effect” where UK cities in the greater London area are entrained to its dynamics. Whereas the US measles data we used extends from 1920-1940, the England and Wales measles data extends from 1944 to 1964.

Our analysis accounts for demographic differences associated with the changing time window between the US and the England and Wales data, including differences in birth rates over time among cities and countries, as detailed below. The temporal mismatch between the US and UK data does not drive the observed epidemic patterns—evidence from other sources clearly shows that measles epidemics in London, UK and other major UK cities remained predominantly biennial in the period covered by the US data (1920-1940; see SI)[15,30].

*Mean periodicity*

Mean periodicity in our analysis is defined as $\int\pi f\left( \pi\right)d\pi$, where $f(\pi)$is the power spectral density of the biweekly time series as a function of period.

*Susceptible reconstruction and parameter estimation for the TSIR model*

On a log scale the dynamics of the infected class in the TSIR model are given by

$$\log E[I_{t+1}]=\log\beta_{t}+\alpha log I_{t}+\log S_{t}-\log N_{t}$$

(S1)

which, once *S_t_* and *I_t_* are reconstructed from case data and birth data (see below), allows parameter estimation within the framework of generalized linear models[11].

Susceptible and infected reconstruction follows standard methods[5]. The equation for the dynamics of the susceptible class is

$$S_{t+1}=S_{t}+B_{t}-\frac{C_{t+1}}{\rho_{t+1}}$$

(S2)

where *B_t_* represents births, *C_t_* represents reported cases, and *ρ_t_* is the time-varying reporting rate. Summing both sides,

$$\sum_{k=0}^{t} S_{k+1}=\sum_{k=0}^{t} S_{k}+\sum_{k=0}^{t} B_{k}-\sum_{k=0}^{t} \frac{C_{k+1}}{\rho_{k+1}}$$

(S3)

shifting indices on the left,

$$\sum_{k=1}^{t+1} S_{k}=\sum_{k=0}^{t} S_{k}+\sum_{k=0}^{t} B_{k}-\sum_{k=0}^{t} \frac{C_{k+1}}{\rho_{k+1}}$$

(S4)

subtracting $\sum_{k=1}^{t} S_{k}$ from both sides,

$$S_{t+1}=S_{0}+\sum_{k=0}^{t} B_{k}-\sum_{k=0}^{t} \frac{C_{k+1}}{\rho_{k+1}}$$

(S5)

and writing the number of susceptible individuals over time in terms of deviations from the long term average

$$S_{t}=\sigma N_{t}+D_{t}$$

(S6)

where σ is the mean proportion susceptible in the population, yields the relation

$$\sum_{k=0}^{t} \frac{C_{k+1}}{\rho_{k+1}}=\sum_{k=0}^{t} B_{k}-D_{t+1}+D_{0}+\sigma\left( N_{0}-N_{t+1} \right)$$

(S7)

from which *ρ_t_* can be estimated by fitting an appropriately smooth monotonic curve to cumulative cases as a function of cumulative births [5]. Mean reporting rate for UK cities was 0.55 (0.06 s.d.) and for US cities was 0.37 (0.13 s.d).

The last two terms in equation S7 have expected value of zero under repeated instances of the analysis in independent stationary populations, meaning it is reasonable to recover *D_t_* from the residual values of the fitted curve. We found that the analysis was more reliable with cases on the left hand side of equation S7 and births on the right, as opposed to the other way around. As cases fluctuate more violently than birth rates, particularly in the US, fitting a curve to cumulative births as a function of cumulative cases resulted in oversmoothing.

The last step to reconstructing susceptible dynamics requires the mean proportion susceptible σ, which has previously been estimated for the England and Wales data by profile likelihood [11]. In order to facilitate comparison across cities we fixed σ at 0.035, to match the proportion susceptible in London, UK. Demonstrably, fixing σ does not damage the analysis. Twenty year forward simulations using the parameterized models yield close fits to the observed data in both deterministic and stochastic simulations (Figure 3). Moreover, σ does not appear in these simulations, which significantly support our most important conclusions (Figures 2-4)—in the forward simulations the number of susceptible individuals is updated iteratively from equation S1, starting from a wide range of initial conditions.

*Lyapunov exponents*

The finite-time dominant Lyapunov exponent for the fitted TSIR model, parameterized from the time series of incidence for a specific city, is given by[6]

 (S8)

where *T* is the number of biweeks used in the calculation, ***J_t_*** is the Jacobian of the deterministic skeleton of the epidemic model (as parameterized for a certain city) and ***U_0_*** *= (1,0)* is a unit vector. We calculated LEs for each city using 100 years (2600 biweekly timesteps) of forward simulations of the deterministic skeleton of the model for each city, following 100 years of “burn-in” to remove the effects of transient dynamics.

*Routes to chaos*

We demonstrate alternate routes to chaos by simulating the TSIR model using synthetic seasonal transmission rate functions for the seasonal transmission rate function *β_t_*_._ These synthetic functions are qualitatively similar to those empirically observed in US and UK (see Fig 2A), but they vary systematically in amplitude or duration, while maintaining constant mean (the global mean of the transmission rates across all cities.)

The synthetic transmission functions are given by

(S9)

where *β^-^* and *β^+^* are the minimum and maximum values attained by the transmission function, *t* measures time in biweeks from January 1 of the current year, and *a* and *b* represent the time of onset and end of the period of low transmission. We then choose *a*, *b, β^-^* and *β^+^*  so the resulting seasonal transmission function has the desired amplitude (*β^+^ - β^-^)* and duration of low *(b-a),* while preserving the mean by adding a scalar constant, once the amplitude and duration are set. Similar to the data, the low period of transmission is always centered at the temporal midpoint of the year.

*Measles epidemics in London, UK and other major UK cities remained predominantly biennial in the period covered by the US data (1920-1940)*

**Figure A.** Annual data (interpolated with a smooth curve) on measles deaths in selected cities in England and Wales[15], showing biennial dynamics in the period covered by the US data.

**Figure B.** (A) Measles incidence patterns across the 20 largest cities in the US and UK respectively. (B) Spatial cross correlations in measles incidence across UK (red) and US (blue) cities. Outer lines enclose bootstrapped 95% confidence intervals on a non-parametric spatial cross correlation function.

**Figure C**: Observed mean periodicity for cities in the UK (crosses) and US (circles) compared to that predicted using the TSIR model parameterized with city-specific fitted seasonal transmission patterns, demonstrating that variation in the shape of the seasonal transmission function can explain a significant portion of the variation in mean periodicity among cities and countries. Colors correspond to mean observed periodicity, as in figures 1 and 4. Simulations were run for 100 years following 100 years of burnin to remove transient dynamics.
